# Validity of the AusTOM scales: A comparison of the AusTOMs and EuroQol-5D

**DOI:** 10.1186/1477-7525-2-64

**Published:** 2004-11-13

**Authors:** Carolyn A Unsworth, Stephen J Duckett, Dianne Duncombe, Alison Perry, Jemma Skeat, Nicholas Taylor

**Affiliations:** 1 School of Occupational Therapy, La Trobe University, Melbourne Vic 3086, Australia; 2 School of Public Health, La Trobe University, Melbourne Vic 3086, Australia; 3 School of Human Communication Sciences, La Trobe University, Melbourne Vic 3086, Australia; 4 School of Physiotherapy, La Trobe University, Melbourne Vic 3086, Australia

**Keywords:** outcomes, assessment

## Abstract

**Background:**

Clinicians require brief outcome measures in their busy daily practice to document global client outcomes. Based on the UK Therapy Outcome Measure, the Australian Therapy Outcome Measures were designed to capture global therapy outcomes of occupational therapy, physiotherapy and speech pathology in the Australian clinical context. The aim of this study was to investigate the construct (convergent) validity of the Australian Therapy Outcome Measures (AusTOMs) by comparing it with the EuroQuol-5D (EQ-5D).

**Methods:**

The research was a prospective, longitudinal cohort study, with data collected over a seven month time period. The study was conducted at a total of 13 metropolitan and rural health-care sites including acute, sub-acute and community facilities. Two-hundred and five clients were asked to score themselves on the EQ-5D, and the same clients were scored by approximately 115 therapists (physiotherapists, speech pathologists and occupational therapists) using the AusTOMs at admission and discharge. Clients were consecutive admissions who agreed to participate in the study. Clients of all diagnoses, aged 18 years and over (a criteria of the EQ-5D), and able to give informed consent were scored on the measures. Spearman rank order correlation coefficients were used to analyze the relationships between scores from the two tools. The clients were scored on the AusTOMs and EQ-5D.

**Results:**

There were many health care areas where correlations were expected and found between scores on the AusTOMs and the EQ-5D.

**Conclusion:**

In the quest to measure the effectiveness of therapy services, managers, health care founders and clinicians are urgently seeking to undertake the first step by identifying tools that can measure therapy outcome. AusTOMs is one tool that can measure global client outcomes following therapy. In this study, it was found that on the whole, the AusTOMs and the EQ-5D measure similar constructs. Hence, although the validity of a tool is never 'proven', this study offers preliminary support for the construct validity of AusTOMs.

## Background

The costs of operating public health services in Australia are rapidly rising. Health administrators and practitioners are under pressure to document client outcomes and demonstrate the effectiveness of therapy interventions [1-3]. Increasingly, the allied health professions have come to see the need for quick, easy to use measures that describe the result of interventions in terms of client outcomes, and provide evaluative data for benchmarking between health service providers [4]. An outcome measure is a tool for documenting change in client status following therapist intervention. This involves the therapist administering a standardized measure at two time points (for example, at admission and at discharge) or at designated time points throughout therapy and then calculating how much change has occurred. The effectiveness of a therapy is shown when the therapist is able to demonstrate that the change in client status was attributable to treatment and not to other factors such as spontaneous recovery [3,5]. In response to the need for outcome measures, a study titled Australian Therapy Outcome Measures (AusTOMs) was funded by the Australian Department of Health and Ageing from 2001–2003. The goal of the study was to develop a reliable and valid measure of therapy outcome for the three largest allied health professions in Australia; occupational therapy (OT), physiotherapy (PT) and speech pathology (SP). The AusTOMs was based on the UK Therapy Outcome Measure (TOM) and adapted and developed to suit the current practices of therapists in Australia [6]. Clinicians can use AusTOMs data which show client change over time in a variety of ways. Clinicians can benchmark their service against other similar facilities which may prompt changes in the type or duration of therapy services offered [4]. Tools such as AusTOMs can also be used in research (such as randomised controlled trials) to evaluate the effectiveness of therapy services.

The original TOM was developed for use by speech and language therapists in the UK for therapists to measure client outcomes in a clinical setting [1]. Later, scales were developed to measure the effects of interventions by occupational therapists, physiotherapists, and rehabilitation nurses [7,8]. Both sets of tools were used to provide benchmarks for therapist practice between service providers [4,8-12]. The development of the TOM was considerably influenced by the International Classification of Impairments, Disabilities and Handicaps 1 and 2 (ICIDH 1&2) [13]. The TOM draws on the ICIDH domains and allows therapists to monitor client status over time in relation to Impairment, Disability, and Handicap. In addition, the developers of TOM added a domain to measure therapist perception of client Wellbeing or Distress (now referred to in this article as Wellbeing). The inter-rater reliability for the four domains of the TOM have been reported for Occupational Therapy as .84 for impairment, .85 for disability, .74 for handicap and .58 for Wellbeing. The reliability for physiotherapists was .66 for impairment, .74 for disability, .77 for handicap and .57 for Wellbeing and for Speech Pathologists the reliability was .89 for impairment, .90 for disability, .84 for handicap and .57 for Wellbeing [1,14].

The AusTOMs was designed to measure client therapy outcome separately for occupational therapists, speech pathologists and physiotherapists. Similar to TOM, AusTOMs provides a 'snapshot' rating that is determined by the clinical judgment of the therapist, which broadly reflects the client's status. The development of the scales and content validity of AusTOMs has been published [6], as has preliminary data concerning the reliability of the scales [15]. Attention has now turned to whether the instrument performs in a manner consistent with the theoretically derived hypotheses underpinning the constructs being measured [16]. The purpose of this paper is to continue the process of validating the AusTOMs, by establishing construct (convergent) validity. Construct validity refers in part to the ability of an instrument to measure an abstract concept or construct. Because constructs are not directly observable and are usually multidimensional, it is important to ascertain that the constructs adequately define and represent the variables that the instrument purports to measure [16]. In particular, convergent validity indicates the degree to which two instruments are measuring similar constructs. Therefore, examination of the construct validity of the AusTOM scales concerns whether the scales actually measure the intended underlying construct of global health-related outcomes.

The researchers attempted to find a 'gold standard' tool to investigate the concurrent validity of the AusTOMs. Health-related quality of life (HRQoL) tools have increasingly been used to assess multiple aspects of health-related quality of life in clinical trials [17]. Tools such as the General Sickness Impact Profile (SIP) [18] measures or infers aspects of activity and participation. The Medical Outcomes Study (MOS) Short Form Health Survey (SF-36) [19] and the Nottingham Health Profile (NHP) [20] measure or infers aspects of impairment, activity and participation. The widely used Functional Independence Measure [21] records the single health domain of activity limitation. However, no tools could be found that measure all four health domains as provided by the AusTOMs, and the tools that were reviewed required too much therapist administration time to be included in the present study. Since there is no gold standard global health status and therapist administered tools with which to compare AusTOMs, it was decided to compare the constructs of AusTOMs with those of EuroQoL-5D (EQ-5D) [22] in order to investigate the convergent validity of the tool [16]. The EQ-5D was chosen for this study since it is widely used in European [22] and Australian studies [23,24], has been used in number of clinical trials [17], is simple and quick to use [25] and similar to the AusTOM, purports to measure global health-related outcomes. However, the potential advantage of using the AusTOMs over the EQ-5D is that while the EQ-5D measures health related outcomes globally, the AusTOMS measures global outcomes in relation to the four specific domains of impairment, activity limitation, participation restriction and wellbeing/ distress. EQ-5D is a short and simple to administer generic HRQoL measure of health status [25]. EQ-5D provides a simple descriptive profile of client problems on five dimensions, an overall score for client self-rated health, and generates a single index value that can be used in the clinical and economic evaluation of health care and in population health surveys [17]. EQ-5D was initially developed in Dutch, English, Finnish, Norwegian and Swedish and is now available in 42 official translations and adaptations [22].

While in principle, health professionals support the notion of measuring health status, there is no consensus regarding the method of measurement [26,27]. While the AusTOMs is rated by therapists, the EQ-5D is rated by the client's themselves. This may be viewed as the main limitation in selecting the the EQ-5D for comparison with the AusTOMS. Nonetheless, it was expected that scores on the AusTOMs scale would vary in relation to scores generated on the EQ-5D since both seek to measure global health-related outcomes. Some researchers prefer the objectivity offered by therapist ratings from observation of client performance [28]. Others support client self-report [29,30] as an accurate reflection of the client's perception of their status, which is becoming increasingly important in consumer-driven heath services. Self -report tools are also considerably cheaper than therapist administered ones, hence, self-report assessments are typically used in a climate requiring cost containment [31]. However, it is also becoming increasingly clear that therapist and client ratings of client performance may not be related [27,32]. In view of the lack of therapist-administered tool suitable to validate the AusTOMs against, and given the time administration advantages of the use of the EQ-5D which were significant to the success of this research program, the EQ-5D was selected for inclusion in the present study.

The purpose of this study was to examine the measurement properties of the AusTOMs and to compare them with the EQ-5D in 'real practice'. The main question being; does AusTOMs perform in a similar manner to the EQ-5D? The study sought to investigate the following hypotheses:

1. There will be a clear pattern of correlations for the admission, discharge and change scores between the AusTOMs domains and the EQ-5D Health Status and Thermometer. Several scale-specific correlations are expected. For example :

a. There will be a moderate negative correlation of the admission, discharge and change scores between the PT AusTOMs Scale 'Pain', Impairment domain and the EQ-5D Health Status Subscale 'Pain'.

b. There will be a moderate negative correlation of the admission, discharge and change scores between OT AusTOMs Scale 'Functional Mobility and Walking', Activity Limitation Domain and the EQ-5D Health Status Subscale 'Mobility'.

c. There will be a moderate negative correlation of the admission, discharge and change scores between OT AusTOMs Scale 'Self-care', Activity Limitation domain, and the EQ-5D Health Status Subscale 'Self-care'.

2. There will be a moderate positive correlation of the admission, discharge and change scores between all the Physiotherapy, Occupational Therapy and Speech Pathology AusTOMs Scales for the Wellbeing /Distress scores and the EQ-5D Thermometer.

## Methods

The research was designed as a prospective, longitudinal cohort study, with data collected over a seven month time period.

### Participants

Thirty-eight occupational therapists, 30 physiotherapists and 47 speech pathologists were trained at 13 participating facilities to collect AusTOMs data, and to present the EQ-5D for clients to complete. However, it is possible that not all these therapists collected data (data collection forms did not require therapists to record their identity). The facilities included acute hospitals, rehabilitation hospitals, and community care facilities. Therapists recorded AusTOMs data and obtained client EQ-5D ratings from 205 clients (110 from Physiotherapy, 67 from Occupational Therapy and 28 from Speech Pathology). These clients were from a larger group of 1007 clients who participated in the study (284 from Physiotherapy, 466 from Occupational Therapy and 257 from Speech Pathology). While some of these participants refused to complete the EQ-5D, or the therapists chose not to burden the client with completing this form, many were children or non-cognizant adults and the EQ-5D is not validated for these groups. Otherwise, the sample was sequential admissions to therapist caseloads over a seven month period.

### Instruments

AusTOMs is comprised of three separate sets of scales for Occupational Therapy (12 scales), Speech Pathology (6 scales) and Physiotherapy (9 scales). The title of each scale is provided in Table 1.

**Table 1:** AusTOMs scales for occupational therapists, speech pathologists and physiotherapists

**Scale**	**Occupational Therapy**	**Speech Pathology**	**Physiotherapy**
1	Learning & Applying Knowledge	Speech	Balance & Postural Control
2	Functional Walking & Mobility	Cognitive-Communication	Cardiovascular System Related Functions
3	Upper Limb Use	Language	Musculoskeletal Movement Related Functions
4	Carrying Out Daily Life Tasks & Routines	Voice	Neurological Movement related Functions
5	Transfers,	Swallowing	Pain
6	Using Transport	Fluency	Respiratory Related Functions
7	Self-care		Sensory functions
8	Domestic Life – Home		Skin functions
9	Domestic Life – Managing Resources		Urinary and bowel continence
10	Interpersonal Interactions & Relationships		
11	Work, employment and Education and Community Life		
12	Recreation, Leisure and Play.		

Each scale requires a rating for four domains of client function, that is, Impairment, Activity Limitation, Participation Restriction and Wellbeing/Distress. An additional optional rating can be made of a caregiver's level of Wellbeing/Distress if the clinician has had contact with a caregiver, and feels that therapy is directed toward the caregiver in some way. Each of the domains are rated by therapists on an 11-point ordinal scale (6 defined points from 0 [most severe] to 5 [normal], and 5 undefined half points). Although clinicians are only required to use the 6 defined scale points, clinicians overwhelmingly chose to include the half points in the AusTOM scoring system to increase scale sensitivity. The use of the half points also facilitates international benchmarking of data against the UK TOM. A generic description of each of the domains of client function is presented in Table 2. Three of the AusTOM's four domains were drawn from the World Health Organisation (WHO)'s International Classification of Function (ICF) [33]. Based on TOM, the AusTOMs were developed by focus groups of expert clinicians in the state of Victoria in Australia who determined both the scale headings, and scalar descriptions for each of the 6 levels for each of the four domains. These scales were then sent out to clinicians across Australia for further refinement. More information on scale development was reported in an earlier publication [6]. In addition, a publication in press [15] reports the reliability of the AusTOM's domains for the majority of scales as ranging from 60–100% agreement, within .5 scalar points for most domains.

**Table 2:** Generic AusTOMs scales (Perry et al, 2004)

***Impairment of either Structure or Function (as appropriate to age):***	
*Impairments are problems in body structure (anatomical) or function (physiological) as a significant deviation or loss*.	
0	The most severe presentation of impairment (either structure or function)
1	Severe presentation of this impairment
2	Moderate/severe presentation
3	Moderate presentation
4	Mild presentation
5	No impairment of structure or function
***Activity Limitations (as appropriate to age):***	
*Activity limitation results from the difficulty in the performanceof an activity. Activity is the execution of a task by the individual*.	
0	Complete difficulty
1	Severe difficulty
2	Moderate/severe difficulty
3	Moderate difficulty
4	Mild difficulty
5	No difficulty
***Participation Restrictions (as appropriate to age):***	
*Participation restrictions are difficulties the individual may have in the manner or extent of involvement in their life situation. Clinicians should ask themselves: "given their problem, is this individual experiencing disadvantage?"*	
0	Unable to fulfill social, work, educational or family roles. No social integration. No involvement in decision-making. No control over environment. Unable to reach potential in any situation.
1	Severe difficulties in fulfilling social, work, educational or family roles. Very limited social integration. Very limited involvement in decision-making. Very little control over environment. Can only rarely reach potential with maximum assistance.
2	Moderately severe difficulties in fulfilling social, work, educational or family roles. Limited social integration. Limited involvement in decision-making. Control over environment in one setting only. Usually reaches potential with maximum assistance.
3	Moderate difficulties in fulfilling social, work, educational or family roles. Relies on moderate assistance for social integration. Limited involvement in decision-making. Control over environment in more than one setting. Always reaches potential with maximum assistance and sometimes reaches potential without assistance.
4	Mild difficulties in fulfilling social, work, educational or family roles. Needs little assistance for social integration and decision-making. Control over environment in more than one setting. Reaches potential with little assistance.
5	No difficulties in fulfilling social, work, educational or family roles. No assistance required for social integration or decision-making. Control over environment in all settings. Reaches potential with no assistance.
**Wellbeing/Distress (as appropriate to age):**	
*The level of concern experienced by the individual. Concern may be evidenced by anxiety, anger, frustration etc*.	
0.	High and consistent levels of distress or concern.
1.	Severe concern, becomes distressed or concerned easily. Requires constant reassurance. Loses emotional control easily.
2.	Moderately severe concern. Frequent emotional encouragement and reassurance required.
3.	Moderate concern. May be able to manage emotions at times, although may require some encouragement.
4.	Mild concern. Able to manage emotions in most situations. Occasional emotional support or encouragement needed.
5.	Able to cope with most situations. Accepts and understands own limitations.

The EQ-5D consists of two parts; the self -classifier or questionnaire, and the EQ-Vas or Thermometer. The EQ-5D self-classifier is a one-page questionnaire, which captures respondent descriptions of health problems on a 5-dimensional classification of mobility, self-care, usual activities, pain and discomfort and anxiety and depression. Each dimension is rated by respondents on a three-level scale from 1 (no problem) to 3 (unable or extreme problem) [22]. The EQ-Vas is a 20-centimeter visual analogue scale, portrayed as similar to a thermometer, on which the respondent rates his/her health state today between 0 (worst imaginable) to 100 (best imaginable). Overall, respondent's health status is either expressed as a score on the visual analogue scale (EQ-Vas), as a profile of their scores on each of the five dimensions (self-classifier), or by combining the scores on the five dimensions. This research utilised the combined scores from the 5 dimensions. The combined dimensions describe 243 theoretically possible health states, that can be converted into a weighted health index score (EQ-Index) for use in cost-effective analysis [26]. The EQ-5D has been shown to be both reliable and valid when used with adult clients with a wide variety of health-related conditions [17,22,25,26].

### Procedure

Approval from the Human Ethics Committee at La Trobe University and the participating facilities was obtained. Study packs were collated for the collection of data. Each pack contained AusTOMs Scale Manual, AusTOMs and EQ-5D data collection forms, informed consent information and consent forms (if these were required by the facility ethics committee). The packs were sent to a contact person in occupational therapy, speech pathology and physiotherapy departments at each site participating in the project. The role of the contact at each site was to receive the packs, disseminate the packs to therapists, check the packs after completion and return them by postage paid envelope.

On admission, the therapists (who had each been previously trained in the use of the scales) briefed each client about the study and after verbal agreement, clients were given a statement of informed consent to read and sign. Clinicians then recorded relevant demographic information and established with the client a specific goal or set of goals for the first episode of care. The therapist then chose the AusTOMs scale/s that best described the main areas targeted for therapy intervention. An admission rating was made by the therapist for each of the four domains of AusTOMs (impairment, activity limitation, participation and wellbeing/distress) on a scale from 0 (most severe) to 5 (least severe). A rating for Wellbeing/ distress was also made for the client's carer if this was applicable to the client's situation. Therapists report that the AusTOMs takes approximately 5 minutes to complete. The therapist then asked the client to complete the self -classifier section of the EQ-5D and the EQ-Vas (Thermometer). Clients were instructed to indicate which statements best described their own health state today, by placing a tick in one box for each of the dimension of mobility, personal care, usual activities, pain/discomfort and anxiety depression. Finally, clients completed the EQ-Vas. Information on the form stated, 'to help people say how good or bad a health state is, we have drawn a scale (rather like a thermometer) on which the best state you can imagine is marked 100 and the worst state you can imagine is marked 0. We would like you to indicate on this scale how good or bad your own health is today, in your opinion. Please do this by drawing a line from the box below to whichever point on the scale indicates how good or bad your health state is today' [22]. Clients completed the EQ-5D in approximately 5 to 20 minutes. The therapist rating for AusTOMs was repeated at client discharge, and clients were asked to again complete both sections of the EQ-5D.

### Data Analysis

The data were analyzed separately for each profession given the differences in the AusTOMs scales. Correlational analyses were performed to investigate the relationship between AusTOMs and EQ-5D. Given the ordinal nature of the scales, a non-parametric approach was adopted, hence all analyses use Spearman's rank-order correlation coefficients (Spearman's Rho). Given the number of correlations performed, alpha (to determine statistical significance) was set at .01, and magnitude of the relationship was considered using the guidelines from Colton [34] where .00 – .25 = little or no relationship, .25 – .50 = a weak to fair relationship, .50 – .75 moderate to good relationship and .76 and above considered good to excellent. In this paper, only relationships that are .5 – .75 (moderate to good), and .76 and above (good to excellent) are reported. In addition, only expected correlations are reported. The optional AusTOMs domain of 'Caregiver Wellbeing' was not included in the analyses since the sample sizes were generally too small to enable computations. Analyses were undertaken across the scales for each profession, and since sample sizes permitted, for the physiotherapy scales: Balance and Postural Control, Musculoskeletal and Neurological, and for the occupational therapy scales: Functional Walking and Mobility, Upper Limb Use, and Self-care.

Sample sizes were not sufficient to enable individual scale analysis for speech pathology scales. The analyses were conducted using only the first scaled selected by the therapist to rate the client. It is also important to note the directions of relationships reported. The EQ-5D Health Status subscales are 1 = no problem -> 3 = unable or extreme problem and the AusTOMs scores are 5 = Normal -> 0 = unable or extreme problem, hence, we expect to see negative correlations. However, the EQ-5D Thermometer scores 0 as the worst state and 100 as the best state and the overall EQ-5D Health Status self classifier score also indicates a better outcome as the score increases, and the AusTOMs scores are 5 = Normal -> 0 = unable or extreme problem. Hence, we expect to see positive correlations between these scores. In the 'Results' the statement is made that the results are in the 'expected direction'.

In line with the research aims and hypotheses, the following analyses were undertaken across each profession's data set. First, a correlation considering all the AusTOMs scales for each domain with EQ-5D Health Status (self classifier score and the 5 dimensions) and Thermometer at admission was performed. Next, AusTOMs scores for each domain for a subset of the most frequently used OT and PT scales with EQ-5D Health Status (self classifier score and the 5 dimensions) and Thermometer at admission were obtained. Then, considering all the AusTOMs scales for each domain were correlated with EQ-5D Health Status (self classifier score and the 5 dimensions) and Thermometer at discharge. Following this, AusTOMs scores for each domain for a subset of the most frequently used occupational therapy and physiotherapy scales were correlated with EQ-5D Health Status (self classifier score and the 5 dimensions) and the Thermometer at discharge. Finally, correlations were obtained for change from admission to discharge scores for AusTOMs (considering all the scales overall and for individual scales) with change from admission to discharge scores for EQ-5D Health Status (self classifier score and the 5 dimensions) and the Thermometer.

## Results

A brief summary of demographic data from the sample is provided in Table 3. The results are presented in relation to the five analyses performed with the data set from each profession. The moderate to good, statistically significant correlations are reported in Table 4 (physiotherapy), Table 5 (occupational therapy), and Table 6 (speech pathology). Rather than present all correlations, only those that would be theoretically expected are presented. In Tables 4, 5, 6, an asterisk is also marked where correlations were expected that were not found, and the sample sizes the analyses were performed on are included since in many cases there is an inadequate sample to detect a relationship.

**Table 3:** Summary of client demographic data

**Variable**	**Occupational Therapy Clients (n = 67)**	**Speech Pathology Clients (n = 28)**	**Physiotherapy Clients (n = 110)**
Mean Age	67.24 (SD 16.65)	64.44 (SD 13.43)	65.44 (SD 20.84)
SEX			
No. Females	41 (61.2%)	11 (39.3.1%)	67 (60.9%)
No. Males	25 (37.3%)	16 (57.1%)	42 (38.2%)
Missing	1 (1.5%)	1 (3.6%)	1 (0.9%)
3 most frequently recorded aetiologies	Acquired neurological 24 (35.8%) Orthopaedic 12 (17.9%) Spinal 6 (9%)	Acquired neurological 14 (50%) Oncology 7 (25%) Neurosurgery 3 (10.7%)	Orthopaedic 44 (40%) Acquired neurological 19 (17.3%) Spinal 9 (8.2%) Musculoskeletal 9 (8.2%)
3 most frequently recorded disorders	Inadequate muscle power 16 (23.9%) Decreased general mobility 11 (16.4%) Multifactorial 11 (16.4%) Pain 9 (13.4%)	Dysphagia (feeding) 9 (32.1%) Acquired language disorder 5 (17.9%) Disorders of voice 5 (17.9%) Dysarthria 4 (14.3%) Cognitive impairment 4 (14.3%)	Abnormal joint mobility 29 (26.4%) Decreased general mobility 24 (21.8%) Inadequate muscle power 15 (13.6%)
SETTING			
No. inpatient	44 (65.7%)	17 (60.7%)	78 (70.9%)
No. outpatient	21 (31.3%)	8 (28.6%)	32 (29.1%)
Missing	2 (3.0%)	3 (10.7%)	
SERVICE TYPE			
Acute	7 (10.4%)	1 (3.6%)	17 (15.5%)
Subacute	49 (73.1%)	24 (85.7%)	68 (61.8%)
Community	9 (13.4%)	2 (7.1%)	15 (13.6%)
Home	0 (0%)	0 (0%)	10 (9.1%)
Missing	2 (3.0%)	1 (3.6%)	
Mean No. of occasions of service	9.05 (SD7.50)	23.28 (SD40.97)	12.36 (SD11.84)

**Table 4:** Summary of Physiotherapy Results: Moderate to strong, statistically significant Spearman's Rho correlations between AusTOMs and EQ-5D

	EQ-5D Therm.	EQ-5D Health status	EQ-5D Mobility Subscale	EQ-5D Self-care Subscale	EQ-5D Usual activities subscale	EQ-5D Pain/ Discom-fort	EQ-5D Anxiety/ depression
AusTOM Over all Impairment							
AusTOM Over all Activity Limitation		*			*		
AusTOM Over all Participation							
AusTOM Over all Wellbeing/ Distress	**0.508** *0.537*						*
AusTOM n = 16 Balance & Pos control Impairment			-0.691 *-0.677*				
AusTOM Balance & Pos control Activity Limitation			*		*		
AusTOM Balance & Pos control Participation							
AusTOM Balance & Pos control Wellbeing/ Distress	*0.655*						-0.739
AusTOM n = 66 Musculoskeletal Impairment							
AusTOM Musculoskeletal Activity Limitation			-0.546		*		
AusTOM Musculoskeletal Participation							
AusTOM Musculoskeletal Wellbeing/ Distress	0.597 **0.614**						**-0.539**
AusTOM n = 18 Neurological Impairment							
AusTOM Neurological Activity Limitation			**-0.801** *-0.746*		*		
AusTOM Neurological Participation							
AusTOM Neurological Wellbeing/ Distress	**0.770**						*

Key: Admission correlation coefficients in normal font

Discharge correlation coefficients in bold font

Change from admission to discharge correlation coefficients in italic

Correlations expected but not obtained marked with *

**Table 5:** Summary of Occupational Therapy Results: Moderate to strong, statistically significant Spearman's Rho correlations between AusTOMs and EQ-5D

	EQ-5D Therm.	EQ-5D Health status	EQ-5D Mobility Subscale	EQ-5D Self-care Subscale	EQ-5D Usual activities subscale	EQ-5D Pain/ Discom-fort	EQ-5D Anxiety/ depression
AusTOM Over all Impairment							
AusTOM Over all Activity Limitation		*			*		
AusTOM Over all Participation							
AusTOM Over all Wellbeing/ Distress	*						-0.612
AusTOM n = 13 Walk & Mobility Impairment							
AusTOM Walk & Mobility Activity Limitation		*	*				
AusTOM Walk & Mobility Participation							
AusTOM Walk & Mobility Wellbeing/ Distress	*						*
AusTOM n = 18 Upper limb use Impairment							
AusTOM Upper limb use Activity Limitation		0.707			*		
AusTOM Upper limb use Participation							
AusTOM Upper limb use Wellbeing/ Distress							
AusTOM n = 16 Self-care Impairment							
AusTOM Self-care Activity Limitation		*0.748*		-0.645 **-0.623** *-0.683*			
AusTOM Self-care Participation							
AusTOM Self-care Wellbeing/ Distress	*						*

Key: Admission correlation coefficients in normal font

Discharge correlation coefficients in bold font

Change from admission to discharge correlation coefficients in italic

Correlations expected but not obtained marked with *

**Table 6:** Summary of Speech Pathology Results: Moderate to strong, statistically significant Spearman's Rho correlations between AusTOMs and EQ-5D

	EQ-5D Therm.	EQ-5D Health status	EQ-5D Mobility Subscale	EQ-5D Self-care Subscale	EQ-5D Usual activities subscale	EQ-5D Pain/ Discom-fort	EQ-5D Anxiety/ depression
AusTOM Over all Impairment							
AusTOM Over all Activity Limitation		*			*		
AusTOM Over all Participation							
AusTOM Over all Wellbeing/ Distress	*						*

Key: Admission correlation coefficients in normal font

Discharge correlation coefficients in bold font

Change from admission to discharge correlation coefficients in italic

Correlations expected but not obtained marked with *

Over all AusTOMs scales for each domain with EQ-5D Health Status (self-classifier score and the 5 dimensions) correlated with the Thermometer at admission (in other words, over all AusTOMs scales for each domain correlated with the EQ-5D Thermometer at admission).

These results are reported in the first 4 rows of Tables 4, 5, 6, normal font. The correlations found, that were expected are all in the expected direction.

AusTOMs scores for each domain for a subset of the most frequently used OT and PT scales with EQ-5D Health Status (self-classifier score and the 5 dimensions) correlated with the Thermometer at admission.

Several moderate to strong correlations were found that were expected and these are presented in Table 4, rows 5–16 for physiotherapy, and Table 5, rows 5–16 for occupational therapy, all in normal font. Again, all correlations were in the expected direction.

Over all AusTOMs scales for each domain with EQ-5D Health Status (self-classifier score and the 5 dimensions) correlated with the Thermometer at discharge.

In relation to this correlation, the results are reported in the first 4 rows of Tables 4, 5, 6, bold font. The correlations found (that were expected) for physiotherapy and occupational therapy were all in the expected direction.

AusTOMs scores for each domain for a subset of the most frequently used OT and PT scales (only) with EQ-5D Health Status (self-classifier score and the 5 dimensions) correlated with the Thermometer at discharge.

The correlations expected that were found are presented in Table 4, rows 5–16 for physiotherapy, and Table 5, rows 5–16 for occupational therapy, all in bold font. Again, all correlations were in the expected direction.

Change from admission to discharge scores for AusTOMs (overall and for individual scales) with change from admission to discharge scores for EQ-5D Health Status (self-classifier score and the 5 dimensions) correlated with the Thermometer.

The moderate to good, statistically significant correlations expected between change on the EQ-5D and AusTOMs overall, or in relation to the six AusTOMs scales where sample size permitted are presented as follows: for physiotherapy (see Table 4, rows 1–16, italic font), occupational therapy (see Table 5, rows 1–16, italic font), and speech pathology (see Table 6, rows 1–4, italic font).

## Discussion

There was some support for the first hypothesis; '...There will be a clear pattern of correlations for the admission, discharge and change scores between the AusTOMs domains and the EQ-5D Health Status and Thermometer (except in relation to the EQ-5D Thermometer and the AusTOMs Wellbeing/ Distress domain as presented in the final hypothesis)'. There were several areas where relationships between constructs measured on AusTOMs and EQ-5D were expected (as described below), and it generally appeared that these two tools are measuring similar constructs. This lends some support to the construct (convergent) validity of AusTOMs. However, not all expected correlations were found and while AusTOMs seems to be measuring global change from the therapist's perspective in relation to four distinct domains (Impairment, Activity Limitation, Participation Restriction and Wellbeing), EQ-5D (as expected), is measuring client perceptions of how they feel about their health status. Hence, while both assessments attempt to capture global health-related outcomes, the differing perceptions of the raters (clinicians versus clients) does seem to impact on the establishment of construct validity. Suggestions for overcoming this problem are described below.

The next sub-hypotheses dealt with specific correlations that were expected in these data. Unfortunately, there were insufficient data to determine if a moderate negative correlation between admission, discharge and change scores between the PT AusTOMs Scale 'Pain', Impairment domain and the EQ-5D Health Status Subscale 'Pain' existed. Similarly, there were insufficient data (n = 13) to explore the hypothesis that '...there will be a moderate negative correlation between admission, discharge and change scores between the OT AusTOMs Scale 'Functional Mobility and Walking', Activity Limitation Domain and the EQ-5D Health Status Subscale Mobility'. The final scale-specific hypothesis predicted a moderate negative correlation between admission, discharge and change scores between the OT AusTOMs Scale 'Self-care', Activity Limitation domain and the EQ-5D Health Status Subscale Self-care. This hypothesis was supported. The results indicate that therapist and client perceptions of client self-care ability status on admission, discharge (and in relation to the change scores) were moderately correlated. Since many occupational therapists spend considerable time working on self-care with clients, and talking about progress in this area, it is reasonable that clients and therapists would rate client status in this area in a similar manner.

Finally, it was hypothesised that there would be a moderate positive correlation between admission, discharge and change scores across all the PT, OT and SP AusTOMs Scales for the Wellbeing domains and the EQ-5D Thermometer. There was only limited support for this hypothesis. However, in some cases the sample sizes were on the small side. There were no moderate, statistically significant correlations when analyzing across all combined OT and SP AusTOMs Scales for the Wellbeing domains and the EQ-5D Thermometer. However, there were moderate to good correlations at both admission and at discharge across all PT AusTOMs Scales for the Wellbeing domains and the EQ-5D Thermometer.

### Study limitations and directions for further research

Given the number of correlations performed for this study, it is important not to over-interpret the relatively small number of moderate and good correlations found. When considering these findings it is also important to note the relatively small sample size since a larger EQ-5D data set may have produced more, significant correlations. The low EQ-5D return rate from speech pathology is not surprising considering that clients seen by speech pathologists often have communication/ cognitive difficulties, and this increases the difficulty in using a self-administered tool such as the EQ-5D. Clinicians also reported that it was difficult to ask quite acutely unwell clients to complete the EQ-5D although they were able to score the client using the AusTOMs.

The validity of a tool is never confirmed. Rather, many studies are required over time to demonstrate that a tool is operating in the manner which developers intended. Future validity studies could investigate the ability of AusTOMs to predict client discharge data from admission status, and to determine the capacity of the tool to discriminate between clients with differing severity levels of impairments and activity limitations. This has already been reported for the physiotherapy profession in relation to the UK TOM [4]. In addition, it would be interesting to compare therapist ratings of clients on the EQ-5D with client ratings on this tool. Such research would provide greater insights to the issue of how similar client and therapist views of clients' health status are. Future validity studies could also compare client data from the AusTOMs with data from other global measures such as the Medical Outcomes Study (MOS) Short Form Health Survey (SF-36) [19] or the Nottingham Health Profile (NHP) [20] measure.

## Conclusion

The EQ-5D is used extensively in cost effectiveness analysis [22]. It is based on client's self report and is thus consistent with the theoretical basis of economic evaluation as the summation of individual utilities. In contrast, AusTOMs are based in therapists' assessment of clinical progress. In the introduction, it was stated that it might be expected that client scores for these two assessments could be different. However, this study revealed that client and therapist assessment appear to be somewhat similar on some domains, thus lending some support for the construct (convergent) validity of AusTOMs. Yet the fact that more, stronger correlations were not found helps to explain some of the differences in perceptions between policy makers, clients, and therapists. Therapists see a range of clients with a given condition and because of their training and experience, have an understanding of what might be achievable in therapy. Clients, on the other hand, make their assessment based on their own experiences and expectations. The differences between client and therapist expectations could perhaps be minimised with better and clearer communication between client and therapist, although neither party in that dyad may be able to accept the inherent limitations of the rehabilitation process. Nonetheless, client perceptions of the success of therapy are vitally important, and more research is required to investigate reasons for the different perceptions of 'therapy success' of these two groups.

The use of different tools across different disciplines to measure improvement can lead to different conclusions about benefits. If outcome measures of cost effectiveness are based on client perceptions it could well be the case that therapy interventions which are seen by therapists to lead to statistically significant improvements in outcome may not be so valued by clients. As a result, those interventions may not be found to be cost effective in an economic sense, if such an evaluation is based on measures of client perception, such as EQ-5D. These differences in perceptions may then contribute to conflict between policy makers, therapists and clients. Alternative economic measures of client outcome, such as return to work, may not be suitable in environments where a significant proportion of clients are beyond working age.

Although the tools appear to be measuring somewhat similar constructs, the results of this study suggest that therapy outcome measures such as AusTOMs may need to be supplemented by client-based measures. As part of the treatment process, differences between responses should be discussed to improve understanding between client and therapist about expectations and achievable outcomes from therapy. This may in turn assist goal setting for the therapy process.

## Authors' contributions

CU and SD have made substantial contributions to conception and design of the study, analysis and interpretation of data and have been involved in drafting the article and revising it critically for important intellectual content. DD has made substantial contributions in the acquisition of data, and has been involved in drafting the article and revising it critically for important intellectual content. APand JS have made substantial contributions to conception and design of the study, acquisition of data, and have been involved in revising the article critically for important intellectual content. NT has made substantial contributions in the acquisition of data and has been involved in revising the article critically for important intellectual content. All authors have given final approval of the version to be published.
